# Soluble monomeric human programmed cell death-ligand 1 inhibits the functions of activated T cells

**DOI:** 10.3389/fimmu.2023.1133883

**Published:** 2023-05-17

**Authors:** Zhaoduan Liang, Wenfang Chen, Yunzhuo Guo, Yuefei Ren, Ye Tian, Wenxuan Cai, Yifeng Bao, Qi Liu, Peng Ding, Yi Li

**Affiliations:** ^1^ Bioland Laboratory, Guangzhou Regenerative Medicine and Health GuangDong Laboratory, Guangzhou, Guangdong, China; ^2^ State Key Laboratory of Respiratory Disease, Guangzhou Institutes of Biomedicine and Health, Chinese Academy of Sciences, Guangzhou, Guangdong, China; ^3^ Division of Life Sciences and Medicine, University of Science and Technology of China, Hefei, Anhui, China; ^4^ T-cell Immunity Optimized Cure (TIOC) Therapeutics Limited, Hangzhou, Zhejiang, China

**Keywords:** peripheral immunity, soluble PD-L1, T cells, exhaustion, peripheral tolerance, cancer, tumor microenvironment

## Abstract

**Introduction:**

The presence of soluble human programmed cell death-ligand 1 (shPD-L1) in the blood of patients with cancer has been reported to be negatively correlated with disease prognosis. However, little information exists about the mechanisms underlying high levels of shPD-L1 for promoting disease progression.

**Methods:**

In this study, we first analyzed the correlations between shPD-L1 and apoptosis of T cells in patients with cancer, then tested the effect of shPD-L1 on T-cell functions and the production of regulatory T cells.

**Results:**

We found that the apoptosis of human peripheral PD-1+CD4+ T cells was significantly elevated in patients with cancer compared with healthy donors and was positively correlated with circulating PD-L1 levels in patients with cancer. In vitro, monomeric shPD-L1 significantly inhibited the proliferation, cytokine secretion, and cancer cell-killing activity of peripheral blood mononuclear cells (PBMCs) activated by either agonist antibodies or HATac (high-affinity T cell activation core)-NYE (NY-ESO-1 antigen). It also promoted CD4+ T cells to express forkhead family transcription factor 3 (FoxP3) for the conversion of induced T regulatory cells, which was more significant than that mediated by soluble human PD-L1 fusion protein (shPD-L1-Fc).

**Discussion:**

These results confirm that soluble PD-L1 could be a candidate for inhibiting the functions of activated T cells, promoting peripheral tolerance to tumor cells, and implicating in system tumor immune escape in addition to the tumor microenvironment. This is an important mechanism explaining the negative correlation between peripheral blood PD-L1 levels and cancer prognosis. Therefore, understanding the roles of hPD-L1 in peripheral blood will be helpful for the development of precision immunotherapy programs in treating various tumors.

## 1 Introduction

Programmed cell death-ligand 1 (PD-L1) is initially discovered as a monomeric membrane-bound ligand (1, 2). The open reading frame of the *PD-L1* gene encodes a protein comprising an extracellular portion, a transmembrane domain, and a cytoplasmic tail (2, 3). PD-L1 is expressed in various tissues and can be detected on dendritic cells, macrophages, regulatory T cells (Tregs), and activated T and B cells (3, 4). PD-L1 is also aberrantly expressed on the surface of human cancer cells (5). Programmed cell death-1 (PD-1) is one of the receptors of PD-L1 that is expressed on activated T cells and Treg cells (4, 6). It is a monomeric type I membrane glycoprotein and a key immune checkpoint molecule (7, 8). The complete PD-1 protein consists of an extracellular immunoglobulin superfamily domain, a transmembrane domain, and a cytoplasmic domain containing two tyrosine-based signaling motifs (8).

The binding of membrane PD-L1 to membrane PD-1 delivers a potent co-inhibitory signal directly to T effector cells, converting T effector cells to induced Tregs (iTregs), which results in T cell exhaustion and dysfunction. This prevents excessive immune response and maintains immune tolerance to self-antigens (9–11). In addition, the ligation of PD-L1 on tumor cells to PD-1 on lymphocytes induces the apoptosis of tumor-specific T cells and promotes the differentiation of CD4^+^ T cells into forkhead family transcription factor 3 (Foxp3)^+^ Tregs. This binding suppresses the killing of tumor cells by cytotoxic lymphocytes within the tumor microenvironment (10, 12, 13).

Recently, soluble PD-L1 (sPD-L1) has been detected in the supernatants of membrane PD-L1-positive cells, including tumor cell lines and immune cells (14, 15). The production of sPD-L1 is related to matrix metalloproteinase enzymes which cleave the extracellular fraction of membrane-type PD-L1 (14) and perform alternative mRNA splicing at the post-transcriptional level (16). sPD-L1 isolated from the supernatants of PD-L1-positive tumor cell lines retains the extracellular PD-1 binding domain, as evidenced through tandem mass spectrometry (17), and its binding activity to the PD-1 receptor (14). Moreover, high levels of circulating PD-L1 are detected in the plasma or sera of patients with hepatocellular carcinoma (18), biliary tract cancer (19), advanced gastric cancer (20), lung cancer (21–23), multiple myeloma (24) and lymphoma (25). The expression of sPD-L1 negatively correlates with cancer prognosis. Patients with high serum/plasma levels of sPD-L1 show an increased mortality risk, while low sPD-L1 levels are associated with a better prognosis (18, 19, 21, 22, 24, 25). However, the underlying mechanisms remain unclear.

Soluble hPD-L1-Fc (shPD-L1-Fc), in which the extracellular domain of hPD-L1 is fused to the crystallizable fragment (Fc) of immunoglobulin to form the hPD-L1 dimer, has been shown to suppress T cell activity (17, 26). Nevertheless, sPD-L1 purified from the serum and culture supernatants of PD-L1-positive tumor cells is observed to exist as a monomer *in vivo* and *in vitro* by evaluating their molecular weights (14, 17, 27). Although shPD-L1-Fc can bind to hPD-1, the physiological functions of recombinant bivalent shPD-L1-Fc may not truly reflect the effect of soluble monomeric human PD-L1 (smhPD-L1) *in vivo*. Therefore, to reveal the correlation between the blood concentrations of smhPD-L1 and cancer prognosis, we analyzed the relationship between the blood concentrations of sPD-L1 and apoptotic PD-1^+^ cells from patients with cancer. In addition, the immune-regulatory activities of smhPD-L1 on human T cells *in vitro* were observed, which were compared with those of shPD-L1-Fc.

## 2 Materials and methods

### 2.1 Patients and healthy donors

A total of 16 patients with hepatocellular carcinoma, 7 patients with cholangiocarcinoma, and 3 patients with pancreatic carcinoma who were newly diagnosed and treated at the Department of Hepatobiliary Surgery, Nanfang Hospital, Southern Medical University, Guangdong, China from September 2017 to November 2017, and 13 healthy donors who served as controls were included in this analysis. Blood samples were drawn prior to any treatment. All patients had histologically confirmed carcinoma. Staging was based on clinical assessment and histopathological analysis according to the International Union Against Cancer (UICC) TNM staging system as recommended by the American Joint Committee on Cancer (AJCC, 7th edition) and the Japanese Gastric Cancer Association (JGCA) guidelines. The Barcelona Clinic Liver Cancer (BCLC) staging system was used to determine the appropriate treatment for patients with hepatocellular carcinoma. The clinicopathological characteristics of the patients were collected from the hospital’s electronic patient records. The study was approved by the Ethics Committee of Guangzhou Institutes of Biomedicine and Health, Chinese Academy of Sciences (approval number: GIBH-IRB2019-006). Informed consent was obtained from each patient and healthy donor.

### 2.2 Cells and cell culture

NCI-H1299 tumor cells (HLA-A*0201^-^ NY-ESO-1^+^) were purchased from the Chinese Academy of Sciences Shanghai Cell Resource Center (TCHu160) in 2010, and Mel624 tumor cells (HLA-A*0201^+^ NY-ESO-1^+^) were kindly provided by Prof. Cassian Yee (MD Anderson Cancer Center, USA). NCI-H1299 tumor cells (HLA-A*0201^+^ NY-ESO-1^+^) were generated via transduction with HLA-A*0201 gene and utilized in this study. The cell lines were treated with M-Plasmocin (InvivoGen) for one week after revival, cultured continuously for less than two months, and authenticated for characteristic markers and growth properties. Mycoplasma testing was performed monthly using PCR. The tumor cells were grown in Dulbecco’s modified Eagle’s medium (DMEM; Gibco) supplemented with 10% heat-inactivated fetal bovine serum (FBS; Gibco) at 37°C in a humidified 5% CO_2_ incubator. Peripheral blood samples from anonymous healthy donors were obtained from the Guangzhou blood center. Peripheral blood mononuclear cells (PBMCs) were isolated via Ficoll-Hypaque gradient centrifugation and maintained in RPMI-1640 medium (Gibco) supplemented with 10% FBS at 37°C in a humidified 5% CO_2_ incubator.

### 2.3 Generation, refolding, and purification of the extracellular region of human PD-L1

Synthetic genes encoding residues 19-229 of *hPD-L1* (NCBI; AY254342.1) were obtained from GenScript to produce soluble monomeric human PD-L1 (smhPD-L1). smhPD-L1 was prepared as previously described (28). Proteins were treated with Triton X-114 to remove endotoxins.

### 2.4 Generation and purification of soluble recombinant Fc fusion protein of hPD-L1 (shPD-L1-Fc)

The gene sequence of human *PD-L1* (NCBI; AY254342.1; residues 19-229) was fused with human *IgG4* (NCBI; P01861; residues 99-327; crystallizable fragment, Fc) to construct *hPD-L1-Fc*. That fusion gene was synthesized and inserted into the *EcoRI* and *NotI* cloning sites of the vector pcDNA3.1(+) (GenScript). The recombinant plasmid pcDNA3.1(+)/hPD-L1-Fc was transfected into HEK293T cells using Lipofectamine 2000 (Invitrogen). The supernatant was collected after culture for three days and purified using an anion QHP column (GE Healthcare) and Superdex™ 200 10/300GL gel filtration column (GE Healthcare). Proteins were treated with Triton X-114 to remove endotoxins.

### 2.5 Expression of HATac-NYE

The high-affinity T cell receptor (TCR) named 1G4 was generated from a wild-type TCR that can recognize a NY-ESO-1_157-165_ peptide presented by HLA-A*0201 (29). The beta chain fused with the anti-CD3 single chain antibody and the alpha chain of 1G4 were expressed in *Escherichia coli* as inclusion bodies. These protein chains were refolded and purified to obtain HATac (high-affinity T cell activation core)-NYE (NY-ESO-1 antigen), as previously described (30, 31).

### 2.6 Analysis of sPD-L1 levels

The serum was obtained from blood samples of 26 patients with cancer and 13 healthy donors, centrifuged (1500 × g, 10 min), aliquoted, and stored at -80°C until analysis. The serum level of sPD-L1 was measured using a commercially available sandwich enzyme-linked immunosorbent assay (ELISA) kit for PD-L1 (USCN Life Science), according to the manufacturer’s protocol. The minimum detectable level of sPD-L1 was 0.066 ng/mL and the detection range was 0.156-10 ng/mL. Samples were measured in duplicates.

### 2.7 Cell assays

For proliferation assays, PBMCs were pre-stained with carboxyfluorescein diacetate succinimidyl ester (CFDA-SE; Molecular Probes) at a final concentration of 1 μM, as described previously (32). Pre-stained PBMCs were stimulated with soluble anti-CD3 (OKT3; 60 ng/mL; BioLegend) and anti-CD28 antibodies (30 ng/ml, BioLegend) in the presence or absence (as a comparable control) of dilute serial concentrations of smhPD-L1 (0.2 μM, 0.4 μM, 1.2 μM, or 2.4 μM) or 0.6 μM of shPD-L1-Fc for four days. For cytokine secretion assays, non-pre-stained PBMCs were stimulated for 48 hours as the proliferation assays. BD GolgiStop™ was added for the last six hours of activation. For the FoxP3 expression assays, PBMCs were activated by soluble OKT3 (60 ng/mL; BioLegend), anti-CD28 antibody (30 ng/mL, BioLegend), and transforming growth factor β (TGF-β; 1 ng/mL; R&D systems) in the presence or absence of smhPD-L1 (0.6 μM, 1.2 μM, or 2.4 μM) or shPD-L1-Fc (0.6 μM or 1.2 μM) for five days. For the lactate dehydrogenase (LDH) and 7-AAD assays, PBMCs were co-cultured with Mel624 or NCI-H1299 cells in the presence of HATac-NYE reagent (1 nM) with or without serial concentrations of smhPD-L1 (0.4 μM, 1.2 μM, or 2.4 μM) or shPD-L1-Fc (0.6 μM) at an E:T ratio of 5:1. After 24 hours of incubation, the supernatant was tested using a CytoTox 96^®^ non-radioactive cytotoxicity kit according to the manufacturer’s instructions (Promega). In addition, the cells were collected and stained with Annexin V PE Apoptosis Detection Kit I (BD Pharmingen), according to the manufacturer’s instructions. For the IncuCyte^®^ assay, PBMCs were co-cultured into flat-bottom plates pre-plated with Mel624 cells (incubated overnight) in the presence of HATac-NYE reagent (1 nM), YOYO^®^-3 Iodide (1:10000 dilution; Thermo Fisher Scientific), and smhPD-L1 (1.2 μM or 2.4 μM respectively) or shPD-L1-Fc (0.6 μM). The plates were incubated in an IncuCyte^®^ ZOOM live cell analysis system for 72 hours. Images were taken every two hour and the number of apoptotic cells per mm^2^ was quantified using YOYO^®^-3 iodide and the IncuCyte^®^ ZOOM software (Essen Bioscience). All conditions were assayed in triplicate.

### 2.8 Fluorescence-activated cell sorting (FACS)

Cell surface and intracellular staining were conducted as previously described (28). The experiments used the following reagents: CFDA-SE, PE-conjugated mouse anti-human IFN-γ (BD Pharmingen), APC-conjugated rat anti-human IL-2 (BD Pharmingen), PE-Cy™7-conjugated mouse anti-human TNF-α (BD Pharmingen), APC-conjugated rat anti-human IL-10 (BD Pharmingen), FITC-conjugated mouse anti-human CD4 (BD Pharmingen), Alexa Fluor^®^ 647-conjugated mouse anti-human Foxp3 (BD Pharmingen), APC-conjugated mouse anti-human CD4 (BioLegend), APC-conjugated mouse anti-human CD8 (BioLegend), PE-conjugated mouse anti-human PD-1 (BD Pharmingen), FITC-conjugated Annexin V (BD Pharmingen), and an Annexin V PE Apoptosis Detection Kit I (BD Pharmingen) with the matched isotype controls. Fluorescence was evaluated via FACS analysis using a BD Accuri™ C6 system within one hour of cell staining. The data were analyzed using FlowJo 7.6 software (Tree Star).

### 2.9 Statistical analysis

Statistical analysis and graphical presentations were performed and generated, respectively, using GraphPad Prism V5.0 software (GraphPad Software Inc.). The data were expressed as the mean ± SEM. Unpaired student’s *t*-test was used to determine the statistical significance between groups, with *p*<0.05 considered significant. Correlations between the serum sPD-L1 levels and phenotypes of apoptotic T cells were analyzed using linear correlation analysis (Pearson’s coefficient). The size of the samples and the number of replicates were given in the corresponding figure legends.

## 3 Results

### 3.1 sPD-L1 levels were significantly positively correlated with the apoptosis of PD-1-positive CD4^+^ T cells

**Figure 1:**
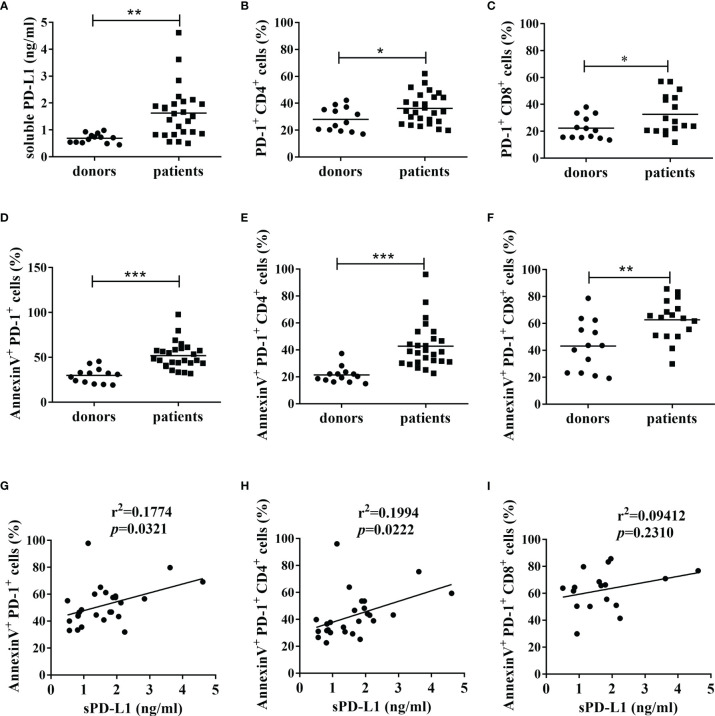
The levels of sPD-L1, PD-1 and apoptosis of PD-1^+^ cells, and their correlations. **(A)** The expression of sPD-L1 from patients with cancer and healthy donors. The serum samples were tested using ELISA. The expression of PD-1 on CD4^+^ cells **(B)** and CD8^+^ cells **(C)** from patients with cancer and healthy donors. The apoptosis level of PD-1^+^ cells **(D)**, PD-1^+^CD4^+^ cells **(E)**, and PD-1^+^CD8^+^ cells **(F)** from patients with cancer and healthy donors. The PBMCs isolated from blood samples were subjected to flow cytometry. Annexin V positivity indicates early apoptosis. Pearson’s correlation analysis between sPD-L1 and AnnexinV^+^PD-1^+^ cells **(G)**, sPD-L1 and AnnexinV^+^PD-1^+^CD4^+^ cells **(H)** in 26 patients with cancer, and sPD-L1 and AnnexinV^+^PD-1^+^CD8^+^ cells **(I)** in 17 patients with cancer. Each dot represents one individual. Unpaired student’s *t*-test, *P<0.05; **P<0.01; ***P<0.001; ns: non-significance. Comparisons are shown in brackets.

To reveal the possible correlation between circulating levels of PD-L1 and the apoptosis of peripheral T cells from patients with cancer, the overall data from 26 patients, of which the baseline characteristics were shown in **Table 1**, and 13 health controls were analyzed. We found that the mean serum PD-L1 levels (**Figure 1A**) and PD-1 positive rate for peripheral CD4^+^ T cells (**Figure 1B**) and CD8^+^ T cells (**Figure 1C**) in patients with cancer were significantly higher than those in healthy donors (*p*<0.01, *p*<0.05 and *p*<0.05, respectively), which were consistent with previous reports (20, 33). Furthermore, the results of flow cytometry demonstrated that the mean apoptosis rate of peripheral PD-1^+^ cells (**Figure 1D**), PD-1^+^CD4^+^ T cells (**Figure 1E**), and PD-1^+^CD8^+^ T cells (**Figure 1F**), as evidenced by Annexin V binding, in patients with cancer was significantly higher than in healthy donors (*p*<0.001, *p*<0.001, and *p*<0.01, respectively). In addition, Pearson’s correlation analysis demonstrated that sPD-L1 expression levels were significantly and positively correlated with the apoptosis of PD-1^+^ cells (r^2 ^= 0.1774, *p*=0.0321, **Figure 1G**) and PD-1^+^CD4^+^ T cells (r^2 ^= 0.1994, *p*=0.0222, **Figure 1H**) in the 26 patients with cancer, but not with that of PD-1^+^CD8^+^ T cells (r^2 ^= 0.09412, *p*=0.2310, **Figure 1I**) in 17 patients with cancer. These results indicate that circulating soluble PD-L1 may correlate with the depletion of peripheral PD-1^+^CD4^+^ T cells in patients with cancer.

**Table 1:** Baseline characteristics of the patient samples (N=26).

Characteristics			Number (%)
Gender	Male		19/26 (73%)
	Female		7/26 (27%)
Age	>60		13/26 (50%)
	≤60		13/26 (50%)
Primary Tumor Classification	Liver cancer		15/26 (58%)
	Cholangiocarcinoma		8/26 (30%)
	Pancreatic cancer		3/26 (12%)
Size (mm)	≤50		18/26 (69%)
	>50		8/26 (31%)
Differentiation	Differentiated	well	3/26 (12%)
		moderate	20/26 (76%)
		poor	3/26 (12%)
	Undifferentiated		0/26 (0%)
Histology	Adenocarcinoma		11/26 (42%)
	Others		15/26 (58%)
Lymph node metastasis	No		22/26 (84%)
	Yes		4/26 (16%)
Distant metastasis	No		25/26 (97%)
	Yes		1/26 (3%)
TNM stage	I		15/26 (58%)
	II		7/26 (27%)
	III		3/26 (12%)
	IV		1/26 (3%)
Tumor Thrombus	Not reported		13/26 (50%)
	No		7/26 (27%)
	Yes		6/26 (23%)
Barcelona liver clinic (BCLC)	A, n (%)		14/15 (93%)
	B, n (%)		0/15 (0%)
	C, n (%)		1/15 (7%)
	D, n (%)		0/15 (0%)

### 3.2 smhPD-L1 suppressed the proliferation of PBMCs

**Figure 2:**
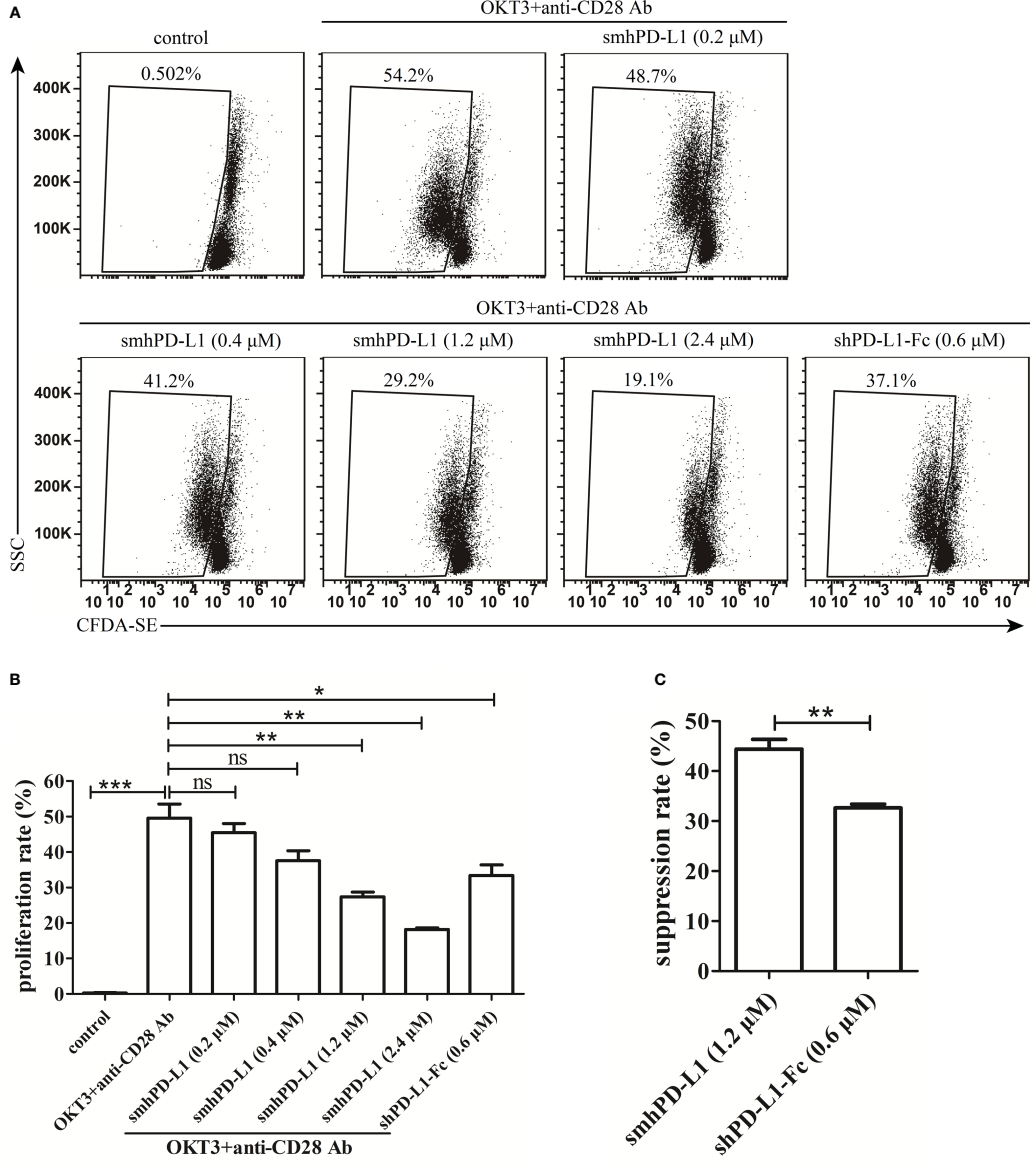
The inhibitory effect of smhPD-L1 or shPD-L1-Fc on the proliferation of PBMCs. **(A)** Representative FACS data on the proliferation of PBMCs. PBMCs pre-stained with CFDA-SE were activated by soluble anti-CD3 and anti-CD28 antibodies in the presence or absence of smhPD-L1 or shPD-L1-Fc for four days. Non-activated PBMCs were used as a negative control. The weakened fluorescence of CFDA-SE, which reflects the proliferation ratio, was detected via flow cytometry. **(B)** Statistical data of the proliferation ratio of PBMCs from **(A)**. **(C)** Comparison of the suppression ratio of PBMCs stimulated with OKT3 and anti-CD28 antibodies in the presence of smhPD-L1 (1.2 μM) or shPD-L1-Fc (0.6 μM). Error bars indicate the SEM (*n*=3). Unpaired student’s *t*-test, *P<0.05; **P<0.01; ***P<0.001; ns: non-significance. Comparisons are shown in brackets. Each experiment was repeated twice.

To determine and compare the effects of smhPD-L1 and shPD-L1-Fc on the proliferation of PBMCs, PBMCs were pre-stained with CFDA-SE. The fluorescence of CFDA-SE could halve in the next generation of cells, indicating cell proliferation. The PBMCs were subsequently stimulated with soluble anti-CD3 antibody (OKT3) in combination with anti-CD28 antibody in the presence or absence of smhPD-L1 or shPD-L1-Fc. As shown in **Figure 2A**, PBMCs significantly proliferated upon stimulation with a combination of anti-CD3/CD28 antibodies; however, their proliferation was inhibited by smhPD-L1 in a dose-dependent manner. A significant inhibitory effect was observed for the group containing 1.2 μM smhPD-L1 (**Figure 2B**). Considering that hPD-L1-Fc is a divalent protein, wherein one molecule of hPD-L1-Fc contains two molecules of hPD-L1, we used equimolar amounts of hPD-L1 to compare the inhibitory effects of smhPD-L1 and shPD-L1-Fc. As shown in **Figure 2C**, the inhibitory rate of shPD-L1-Fc was 33%, which was lower than that of smhPD-L1 (44%; *p*<0.01). Therefore, non-immobilized smhPD-L1 demonstrated similar inhibitory effects as those of immobilized PD-L1-Fc on plastic plates or beads (6, 9), and the inhibition effect of smhPD-L1 on the proliferation of PBMCs was stronger than that of shPD-L1-Fc.

### 3.3 smhPD-L1 inhibited the secretion of cytokines

**Figure 3:**
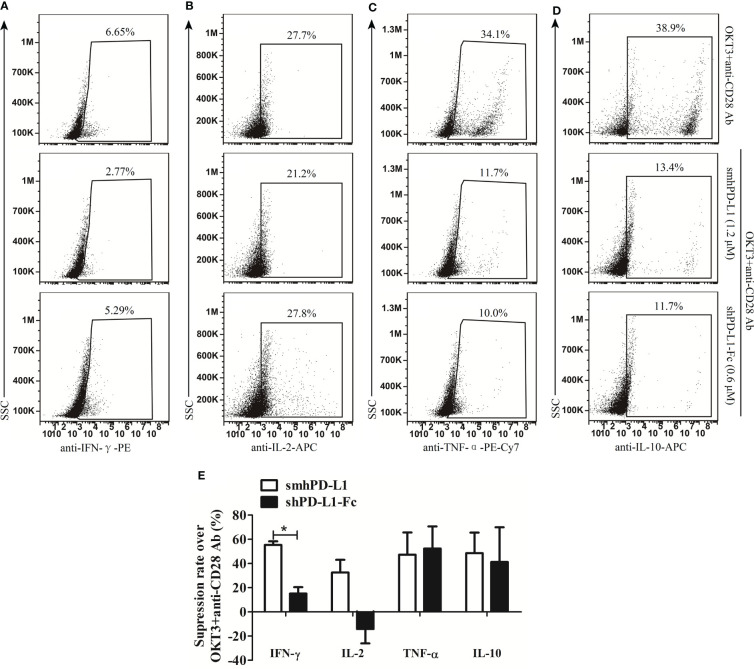
The effects of smhPD-L1 and shPD-L1-Fc on the cytokine secretion of PBMCs. PBMCs were activated with soluble anti-CD3 and anti-CD28 antibodies in the presence or absence of smhPD-L1 or shPD-L1-Fc for 2 d. Representative FACS data on the secretion of IFN-γ **(A)**, IL-2 **(B)**, TNF-α **(C)**, and IL-10 **(D)** after staining intracellularly and detecting via flow cytometry. **(E)** Statistical analysis on the suppression rates from **(A–D)**. The analysis compared the capability of smhPD-L1 and shPD-L1-Fc in suppressing the expression of IFN-γ, IL-2, TNF-α, and IL-10 in combination with anti-CD3/CD28 antibodies to activate T cells. Error bars indicate the SEM (*n*=3). Unpaired student’s *t*-test, *P<0.05. Comparisons are shown in brackets. Each experiment was repeated twice.

To evaluate the inhibitive effects of smhPD-L1 and shPD-L1-Fc on cytokines production, smhPD-L1 and shPD-L1-Fc were supplemented to the PBMCs stimulated with anti-CD3/CD28 antibodies. The intracellular expression levels of IFN-γ, IL-2, TNF-α, and IL-10 in PBMCs were analyzed. IFN-γ levels were reduced by 55.3% upon supplementation of smhPD-L1, while only a 15.1% reduction was observed for shPD-L1-Fc supplementation (*p*<0.05, **Figures 3A, E**), which was lower than that with pre-coated PD-L1-Fc (6, 9). IL-2 levels in the PBMCs revealed a 32.5% reduction upon treatment with smhPD-L1; however, IL-2 levels increased by 14.2% after treatment with shPD-L1-Fc (**Figures 3B, E**). After supplementation with smhPD-L1 or shPD-L1-Fc, the reductions in TNF-α levels in the PBMCs (47.3% and 52.4%, respectively; **Figures 3C, E**), as well as the reduction of IL-10 levels in the PBMCs (48.5% and 41.3%, respectively; **Figures 3D, E**), were not significantly different. Thus, compared to divalent shPD-L1-Fc, smhPD-L1 demonstrated superior IFN-γ and IL-2 suppression, while there was no significant difference in the suppressive capacity between these two PD-L1 molecules on TNF-α and IL-10 production in PBMCs.

### 3.4 smhPD-L1 inhibits the tumor cell-killing capacity of PBMCs as redirected by HATac-NYE

**Figure 4:**
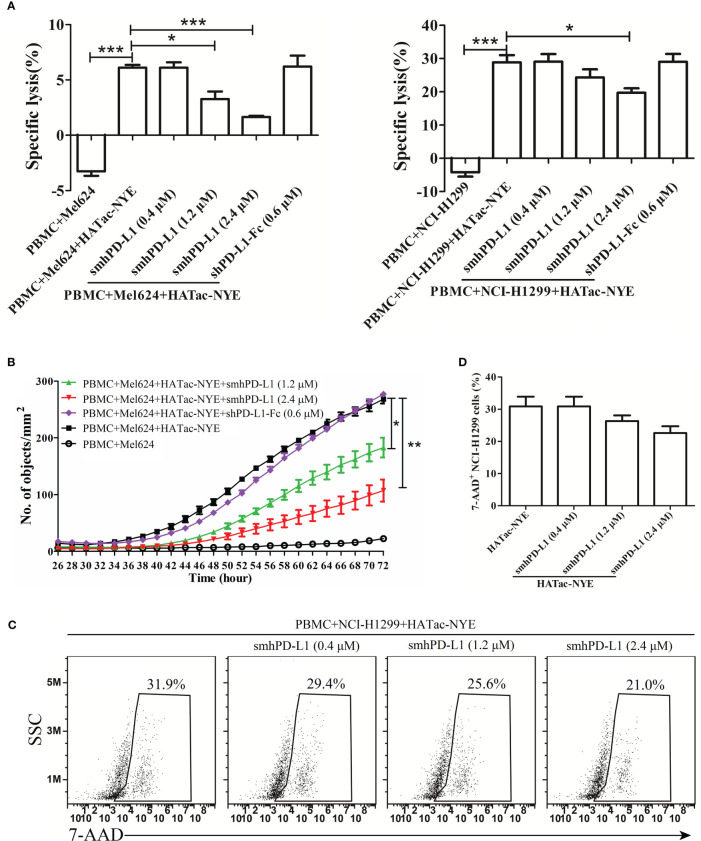
smhPD-L1 inhibits PBMCs from killing tumor cells as induced by HATac-NYE. **(A)** Specific lysis of Mel624 cells and NCI-H1299 cells was evaluated through the release of LDH. The lysis of Mel624 cells and NCI-H1299 cells by PBMCs was directed by HATac-NYE (1 nM) in the presence or absence of different concentrations of smhPD-L1 (0.4 μM, 1.2 μM, or 2.4 μM) or shPD-L1-Fc (0.6 μM) at a 5:1 E:T ratio for 24 h. **(B)** The apoptosis of Mel624 cells was evaluated through the intracellular levels of YOYO^®^-3 iodide. Mel624 cells were co-cultured with PBMCs in the presence of HATac-NYE (1 nM) combined with different concentrations of smhPD-L1 (1.2 μM or 2.4 μM) or shPD-L1-Fc (0.6 μM) at a 5:1 E:T ratio for 72 h. The culture system was supplemented with YOYO^®^-3 iodide, whose fluorescence signal was detected using an IncuCyte^®^ ZOOM live cell analysis system. **(C)** The apoptosis of NCI-H1299 cells was evaluated using the positivity ratio of 7-AAD. NCI-H1299 cells were co-cultured with PBMCs in the presence of HATac-NYE (1 nM) combined with different concentrations of smhPD-L1 at a 5:1 E:T ratio for 24 h. All cells were stained with 7-AAD, NCI-H1299 cells were gated, and the fluorescence signal of 7-AAD was detected via flow cytometry. **(D)** Statistical analysis of the 7-AAD positivity rate in NCI-H1299 cells from **(C)**. Error bars indicate the SEM (*n*=3). Unpaired student’s *t*-test, *P<0.05; **P<0.01; ***P<0.001. Comparisons are shown in brackets. Each experiment was repeated twice.

To investigate the effect of smhPD-L1 on tumor cell growth under immunological surveillance, we set up an *in vitro* system using a reagent named HATac-NYE to monitor the tumor cell-killing ability of PBMCs. HATac-NYE was previously demonstrated to have potent efficacy for redirecting T cells to lyse tumor cells *in vitro *(30). smhPD-L1 inhibited HATac-NYE to mediate the PBMC-induced lysis of Mel624 and NCI-H1299 tumor cells in a dose-dependent manner. In particularly, 2.4 μM of smhPD-L1 had a significant inhibitory effect, as demonstrated by the release of LDH (**Figure 4A**). We used an IncuCyte^®^ ZOOM live cell analysis system to observe the lysis of Mel624 tumor cells in real-time for 72 h after treatment with YOYO^®^-3 iodide. smhPD-L1 significantly suppressed the intracellular fluorescence of YOYO^®^-3 iodide in Mel624 tumor cells in a dose-dependent manner from 38 to 72 h (**Figure 4B**). Furthermore, using an assay involving HATac-NYE to redirect PBMCs and produce apoptotic tumor cells, smhPD-L1 decreased the apoptosis of NCI-H1299 tumor cells in a dose-dependent manner, as evidenced by positive 7-AAD staining at 24 h (**Figures 4C, D**). To our surprise, as shown in **Figures 4A, B**, at an equimolar quantity, divalent shPD-L1-Fc did not significantly affect the release of LDH and the intracellular fluorescence of YOYO^®^-3 iodide in tumor cells. These results confirmed that smhPD-L1, but not divalent shPD-L1-Fc, inhibited the capacity of HATac-NYE-redirected PBMCs to kill tumor cells.

### 3.5 smhPD-L1 promoted Foxp3 expression in CD4^+^ T cells

**Figure 5:**
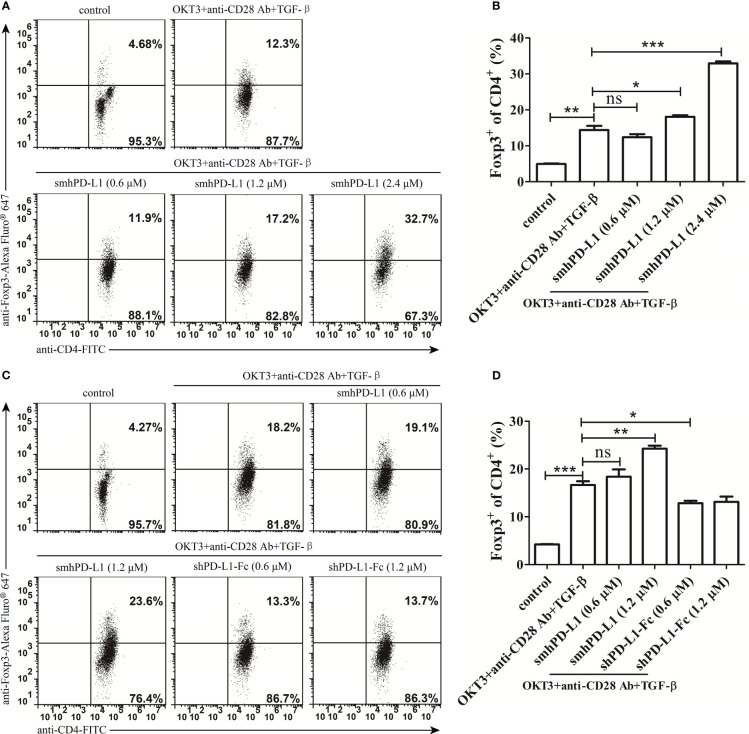
smhPD-L1 mediates Foxp3-induced Treg cell development. **(A)** Representative FACS data of smhPD-L1 showing the promotion of CD4^+^ T cells to express Foxp3. PBMCs were activated with soluble anti-CD3-antibody (OKT3, 60 ng/mL), anti-CD28-antibody (30 ng/mL), and TGF-β (1 ng/mL) in the presence of different concentrations of smhPD-L1 (0.6 μM, 1.2 μM, or 2.4 μM) for 5 d. Non-activated PBMCs were used as a negative control. **(C)** Representative FACS data comparing the effect of smhPD-L1 and shPD-L1-Fc on Foxp3 expression. PBMCs were activated as previously described and incubated in the presence of smhPD-L1 or shPD-L1-Fc for 5 d. Non-activated PBMCs were used as a negative control. Flow cytometry was used to analyze the fluorescence of CD4-FITC- and Foxp3-Alexa Fluor^®^ 647-stained PBMCs. **(B)** Statistical data of the Foxp3 positivity ratio in CD4^+^ T cells from **(A)**. **(D)** Statistical data of the Foxp3 positivity ratio in CD4^+^ T cells from **(C)**. Error bars indicate the SEM (*n*=3). Unpaired student’s *t*-test, *P<0.05; **P<0.01; ***P<0.001; ns: non-significance. Comparisons are shown in brackets. Each experiment was repeated twice.

To investigate the effect of smhPD-L1 on CD4^+^ T cells expressing Foxp3, a key molecule for the differentiation and function of Tregs (11, 34), freshly isolated PBMCs were stimulated using anti-CD3 antibody (OKT3, 60 ng/mL), anti-CD28 antibody (30 ng/mL), and TGF-β (1 ng/mL) with or without smhPD-L1 or shPD-L1-Fc for five days. We found that smhPD-L1 significantly promoted CD4^+^ T cells to express Foxp3 in a dose-dependent manner (**Figures 5A, B**), indicating that smhPD-L1 could promote the conversion of Tregs from CD4^+^ T cells *in vitro*. The conversion effect of smhPD-L1 (1.2 μM) on Tregs was significant (*p*<0.05) (**Figure 5B**); nevertheless, shPD-L1-Fc (0.6 μM), which contained the same molar amount of PD-L1 with smhPD-L1 (1.2 μM), significantly inhibited the expression of FoxP3 (*p*<0.05). Its inhibitory effect was maintained at 1.2 μM of shPD-L1-Fc (**Figures 5C, D**), contrary to that obtained using the immobilized form of PD-L1-Fc (11). This result suggests that only a 1:1 combination of PD-L1 to PD-1, similar to its membrane-binding mechanism (1), truly reflects the physiological functions of the PD-1 axis on the immune system.

## 4 Discussion

Immunotherapeutic approaches could often initiate T cell responses in experimental animal models loaded with tumors and patients with cancer. However, these responses are often not correlated with tumor regression in clinical trials (35), which has been traditionally attributed to the tumor microenvironment. Tumor cells can reprogram the tumor microenvironment, forming a strong immunosuppressive network to limit the ability of T cells to eradicate tumor cells (36). The tumor microenvironment consists of tumor cells, associated stromal cells, infiltrating immune cells, the surrounding extracellular matrix, and interstitial fluid (36). PD-L1 is initially discovered as a membrane protein on the cell surface and is expressed in various cells, including tumor tissues (3, 12). It is well-known that the interaction of membrane PD-L1 to membrane PD-1 directly affects TCR signals by inhibiting the phosphorylation of the ZAP70, PI3K-Akt, and Ras-MEK-ERK pathways. This dephosphorylation results in T cell exhaustion, one of the mechanisms tumor can silence immune surveillance in the tumor microenvironment (37).

Functional decline and a higher expression of inhibitory receptors (such as PD-1) are some hallmarks of exhausted T cells (38). In some cases, exhaustion can be manifested by clonal deletion, which results in the physical removal of effector cells from the immune repertoire (39). Interacting with tumor-associated PD-L1, activated T cells are led to programmed cell death through the upregulated expression and interaction of Fas and FasL between activated T cells (12). The rapid increase in the proportion of apoptotic T cells after co-culture with PD-L1-transfected tumor cells suggests an *in vivo* deletion of activated T cells by tumor-associated PD-L1 in tumors (12). Recently, high levels of circulating sPD-L1 are detected in the peripheral blood of various patients with cancer (18, 20–25). Our results demonstrated that, except for the upregulation of sPD-L1 (**Figure 1A**), PD-1 expression on peripheral CD4^+^T cells (**Figure 1B**), and the apoptotic rate of peripheral PD-1^+^cells (**Figure 1D**) and PD-1^+^CD4^+^T cells (**Figure 1E**) were significantly increased in patients with cancer compared with healthy donors. Moreover, sPD-L1 expression levels were significantly positively correlated with the apoptosis of PD-1^+^cells and PD-1^+^CD4^+^T cells (**Figures 1G, H**). These results indicate that sPD-L1 may correlate with the *in vivo* deletion of activated T cells in the peripheral circulation, which may modulate the immune response in peripheral tissues, and participate in the negative correlation between circulating sPD-L1 levels and cancer prognosis.

In order to further reveal the correlation between sPD-L1 and dysfunctional T cells, we refolded the extracellular domain of hPD-L1 from inclusion bodies *in vitro* to obtain soluble monomeric hPD-L1 (smhPD-L1), and detected its effect on T-cell functions. Our results showed that smhPD-L1 inhibited the anti-CD3 antibody-activated PBMCs from secreting IL-2 (**Figure 3B**), which interacts with the IL-2 receptor complex, promotes proliferation of T cells (40), and related to the proliferation of PBMCs activated by anti-CD3 antibody was significantly inhibited by smhPD-L1 (**Figures 2A, B**). In addition, smhPD-L1 inhibited anti-CD3 antibody-activated PBMCs from secreting IFN-γ (**Figure 3A**) which directly enhances the cytotoxicity of cytotoxic lymphocytes (41), TNF-α (**Figure 3C**) which rapidly induces target cells expressing apoptosis-related receptors (42), and IL-10 (**Figure 3D**) which induces the cytotoxic activity of CD8^+^T cells (43). Those results related to that smhPD-L1 inhibited HATac-NYE-redirected PBMCs from killing NY-ESO-1/HLA-A*0201-positive tumor cells (**Figure 4**). Furthermore, the suppressive effect of shPD-L1-Fc on the secretion of IFN-γ was significantly weaker compared to smhPD-L1 (**Figure 3A**). shPD-L1-Fc also promoted PBMCs to secrete IL-2 in the presence of anti-CD3 antibody (**Figure 3B**), showing that the inhibitory effects of shPD-L1-Fc on cell proliferation and cytotoxicity were weaker than those of smhPD-L1 (**Figures 2C**, **4A, B**). Those resulted from that bivalent shPD-L1-Fc has a stronger binding strength to PD-1 than monomeric hPD-L1, and that the inhibition effect of the hPD-1 signaling pathway on the TCR-CD3 signaling pathway decreases with increasing binding strength (28). Our results suggest that monomeric hPD-L1 in solution or on the cell membrane has similar inhibitory functions on T effector cells, which is related to dysfunctional peripheral T cells through the loss of their effector functions.

Acquiring immunity to cancer is defined as the Cancer-Immunity Cycle. Each step of this cyclic process requires the coordination of numerous stimulatory and inhibitory factors, including membrane PD-L1 expressed on DCs and the tumor bed (44). PD-1 is found to be highly expressed in peripheral effector T cells from patients with renal cell carcinoma (45) or non-small cell lung cancer (33). The expression of PD-1 on T cells is increased after priming with antigen presentation cells (46). High levels of circulating sPD-L1 are also detected in the plasma or sera of patients with tumors (18). Upon binding to its ligands, the PD-1 axis initiates suppressive signals to inhibit proximal TCR signaling events, which results in the decreased proliferation, cytokine production, and cytolytic capability of T cells (34). Our results had shown that, upon binding to PD-1, smhPD-L1 significantly suppressed T cell proliferation, cytokine production, and cytolytic capability. And so on, in patients with cancer, the pre-existence of the smPD-L1/PD-1 interaction on T cells inhibits DCs from priming T cells, which home to the lymph nodes from the peripheral blood, then traffic and infiltrate into the tumor microenvironment, and has their tumor cell-killing ability suppressed. Therefore, it is expected that PBMCs isolated from the peripheral blood of patients with lung cancer are poorly redirected by ImmTAC-NYE from killing tumor cells (47).

In addition to negatively regulating conventional T-cell functions, the interaction of PD-L1 on the surface of APCs or beads with PD-1 expressed on CD4^+^T cells contributes to peripherally-induced Treg cell (piTreg) development by suppressing Akt-mTOR signals and enhancing and maintaining the Foxp3 expression of piTreg, which is critical for maintaining peripheral tolerance (10, 11). Our results showed that smhPD-L1 significantly promoted anti-CD3 antibody and TGF-β stimulated CD4^+^ T cells to express Foxp3 in a dose-dependent manner *in vitro* (**Figures 5A, B**), showing a similar trend as PD-L1 coated on beads (11). High levels of circulating PD-L1 (**Figure 1A**) and PD-1 expressed on peripheral CD4^+^ T cells (**Figure 1B**) were detected in patients with cancer, as previously reported (33). Our results (**Figures 5A, B**) suggest that high levels of sPD-L1 might convert peripheral PD-1^+^CD4^+^ T cells to circulating iTregs, which are detected in several studies (48–51). Moreover, the interaction between PD-1 on Tregs and PD-L1 is necessary to exert the strong suppressive effect of Tregs on effector T cells (52). Our results showed that smhPD-L1 in peripheral blood had a similar effect on the PD-1 axis for conversing Tregs. Therefore, we speculated that shPD-L1 might contribute to the suppressive functions of circulating Treg cells in patients with cancer, which could promote peripheral tolerance to tumor cells and be implicated in tumor immune escape outside the tumor microenvironment.

In conclusion, our study provides fundamental evidence showing that levels of circulating sPD-L1 are significantly positively correlated with the apoptosis of peripheral CD4^+^T cells in patients with cancer. smhPD-L1 not only negatively regulates the proliferation, cytokine secretion, and cytolytic capability of activated T cells, but also promotes Treg-cell generation, which systemically suppresses the cytotoxic immune response. Combined with recent reports regarding the correlation between high levels of peripheral PD-L1 and poor cancer prognosis, our results suggest that circulating PD-L1 can act as an additional tumor immune suppressive mechanism outside the tumor microenvironment for cancer progression. Therefore, it is important to design precision therapies for patients with cancer who have high levels of sPD-L1, considering the sPD-L1 immune escape mechanism for successful treatment. Specifically, we should think carefully about using PD-1 axis blockade therapy to treat patients who have high levels of peripheral PD-L1.

## Data availability statement

The original contributions presented in the study are included in the article/supplementary material. Further inquiries can be directed to the corresponding author.

## Ethics statement

Written informed consent was obtained from the individual(s) for the publication of any potentially identifiable images or data included in this article.

## Author contributions

YL and ZL designed the study. ZL collected and analyzed the blood samples. ZL, WFC, YG, YR, YT, WXC, YB, QL, and PD performed the immunological experiments. YT conducted the statistical analysis. YL and ZL analyzed and interpreted the data. ZL and YL wrote the manuscript. All authors contributed to the article and approved the submitted version.
